# Synergistic Antioxidant and Antibacterial Advantages of Essential Oils for Food Packaging Applications

**DOI:** 10.3390/biom11091267

**Published:** 2021-09-02

**Authors:** Nagaraj Basavegowda, Kwang-Hyun Baek

**Affiliations:** Department of Biotechnology, Yeungnam University, Gyeongsan 38451, Korea; nagarajb2005@yahoo.co.in

**Keywords:** essential oils, antioxidant, antibacterial, synergistic, food packaging, food additives

## Abstract

The development of food-borne and infectious diseases has increased globally at an anomalous rate and is combined with emerging social and economic problems. This highlights the need for new and improved antibacterial agents with novel and different mechanisms of action at regular intervals. Some chemical or artificial food additives are considered harmful if they are used beyond their permissible levels. Today, consumers are demanding alternative, green, safer, and natural food additives to increase the shelf life of food. Essential oils (EOs) are concentrated liquid mixtures of volatile compounds with antioxidant and antibacterial properties that can be used as natural, eco-friendly, renewable, and cost-effective additives. The use of combinations of different EOs and their components is a promising strategy to increase the synergistic and additive effects of EOs in foods. In this article, we review the recent literature on EOs concerning the chemical constituents, extraction methods, antioxidant and antibacterial activities, and their mechanisms of action. Additionally, we discuss the synergistic interaction of different EOs and their components, challenges, and future directions of EOs as natural food preservatives, with special emphasis on shelf life extension and applications in the packaging of food products.

## 1. Introduction

Microbial food spoilage and oxidative deterioration during the storage of foods are major concerns for our society. Pathogenic microorganisms, including bacteria and fungi, spoil perishable foods, such as fruits, vegetables, fish, meat, poultry, and fresh cereal-based products, and generally cause changes in flavor, texture, color, odor, and taste [1]. In addition, enzymes, air, light, and temperature cause changes in color, texture, and flavor, and reduce the shelf life of foods, thereby increasing the risk of foodborne illness. As a result, artificial or synthetic preservatives have been used by the food industry for many years. These artificial preservatives are chemical substances such as nitrites, benzoates, propionates, and sorbates, which destroy bacteria or inhibit the growth of mold on foods. Similarly, sulfites, tocopherol, ascorbic acid, butylated hydroxyanisole, and butylated hydroxytoluene are antioxidants that inhibit oxidation and disodium ethylenediaminetetraacetic acid, citric acid, and polyphosphates are chelating agents that can slow down or restrict the deterioration of food [2]. However, it is very important for consumers to be aware of some synthetic preservatives that are toxic, carcinogenic, and commonly used at high concentrations [3]. Thereby, it is essential that consumers purchase safer, high-quality, natural, and less processed foods that are free from synthetic preservatives and have extended shelf lives. This situation has motivated scientists to explore natural alternatives to chemical or synthetic food preservatives.

Floral waters or hydrosols are mixtures of water-soluble compounds of the plants and trace amounts of EOs, obtained as a by-product of the steam distillation process. When plant materials are subjected to distillation and EOs of plant material are drawn off, the remaining water still contains certain water-soluble constituents of the plant material, which are free from the lipophillic substances known as floral waters or hydrosols. Floral waters are used as air freshener, cooling agents, body sprays, deodorants, facial spritzes, etc., Rose, lavender, eucalyptus, helichrysum, neroli, lemongrass, tea tree, and chamomile floral waters are the most popular examples.

Packaging systems play a primary role in containing and adequately protecting food products as they move through the supply chain to the consumer. The use of proper packaging methods and materials to maintain/provide high quality, superior taste, fresh, safe, and convenient food products have constantly increased in recent decades [4]. In addition, packaging meets the fundamental need to extend the shelf life of food by preventing unwanted chemical and biological changes from production to final consumption. Consequently, changes in retail practices, globalization of markets, centralization of activities, and longer transport distances have become major challenges for food packaging industries in designing supply chains and moving food products to consumers [5]. The main function of packaging is to provide passive protection to the products or act as a barrier between the food, atmosphere, and the external environment [6]. Some properties of the additives with active functions in food packaging systems include absorbing/scavenging properties (additives include moisture, oxygen, carbon dioxide, ethylene, flavors, and UV light); releasing/emitting properties (e.g., antioxidants, preservatives, sulfur dioxide); removing properties (e.g., lactose and cholesterol); and temperature, and microbial control [7].

Several diseases, such as cancer and neurodegenerative diseases, as well as deterioration of food and meat have been attributed to free radicals. The use of antibiotics creates a favorable environment for the growth of multidrug-resistant (MDR) bacteria, which is another problem affecting public health. Some MDR bacteria, especially methicillin-resistant *Staphylococcus aureus*, penicillin-resistant *Streptococcus pneumoniae*, fluoroquinoline-resistant *Pseudomonas aeruginosa*, multi-antibiotic resistant *Acinetobacter baumannii*, ceftazidime-resistant *Klebsiella pneumoniae*, and *Escherichia coli* are acquired pathogens [8,9]. In addition, *Bacillus cereus*, *E. coli*, *Clostridium perfringens*, *C. botulinum*, *Campylobacter jejuni*, *Cronobacter sakazakii*, *Listeria monocytogenes*, *S. aureus*, *Yersinia enterocolitica*, *Salmonella enteritidis*, *Shigella dysenteriae*, and *Vibrio furnissii* as food-borne pathogens cause various illnesses and have a major impact on public health [10]. The spread of MDR bacteria makes it imperative to identify new classes of antimicrobials and compounds that inhibit resistance mechanisms. Currently, the use of natural antimicrobial compounds in food has received great attention among consumers and the food industry because of their economic viability, low toxicity, and pharmacological activities. In addition, natural products have attracted much attention owing to their wide availability and better biodegradability, and their potential as alternatives to synthetic preservatives [11]. Consequently, plants, plant-extracted phytochemicals, and essential oils (EOs) are used as antimicrobial agents for the prevention of foodborne pathogens and spoilage bacteria by direct addition to food commodities or for use in synergy with other EOs or their constituents [12].

Among natural antimicrobials, EOs have been widely used as food flavors because of their antibacterial, antifungal, antioxidant, antiseptic, anti-inflammatory, anticarcinogenic, and antimutagenic properties. EOs are liquid mixtures of volatile and hydrophobic compounds obtained from different parts of aromatic medicinal plants, such as leaves, buds, flowers, shoots, peels, barks, twigs, fruits, seeds, and roots. They provide the essence of the plant with specific odoriferous and lipophilic characteristics, which are responsible for the aroma and flavor of spices [13]. Currently, approximately 3000 varieties of EOs are well known, of which approximately 300 are commercially important in the flavor, fragrance, pharmaceutical, food, and cosmetic industries [14]. Moreover, it is well known that plants produce a wide variety of secondary metabolites with remarkably low boiling points that influence the oxidative stability of EOs and have good antioxidant properties to protect food from rancidity [15]. EOs are secreted by specialized secretory plant tissues called glandular trichomes, which are multicellular epidermal glands that diffuse onto the surface of plant organs, particularly leaves and flowers [16]. EOs secreted by trichomes are a mixture of several low molecular weights (<300 Da) and other volatile compounds, including terpenoids, phenylpropanoids, isoprenoids, phenols, alcohols, and aldehydes [16].

The amount of EOs varies among different components, parts of plants, and plant species, and this determines the price of EOs. The main EOs responsible for antimicrobial and antioxidant properties are thyme, lemon, clove, cinnamon, and tea tree oils, which effectively increase the quality and shelf life of food and other cereal products [17]. The antimicrobial action of EOs is mainly a consequence of their hydrophobic nature, which enables them to partition into the lipid layer cell membrane and mitochondria, providing them with greater permeability, causing damage and disrupting the cell wall structures [12]. Thus, EOs are promising alternative compounds to serve as natural additives, preservatives, and active packaging systems in food and food products to reduce the existing problems associated with food safety risks from chemically synthesized additives [18]. In this review, we present the antibacterial and antioxidant activities with mechanisms of action, chemical constituents, and the methods of extraction of EOs. Furthermore, we have discussed the synergistic interaction of different EOs and their constituents and shelf-life extension of packaged food with current trends and future perspectives.

## 2. Chemical Constituents of Essential Oils

**Figure 1:**
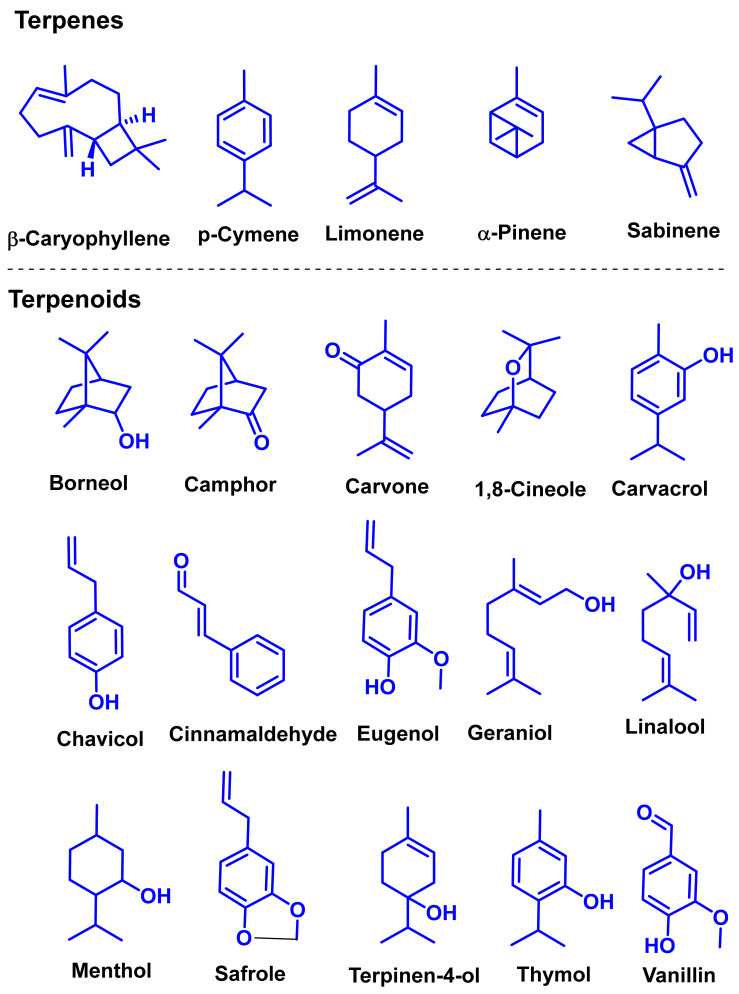
Chemical structural formulas of some major constituents of essential oils.

The chemical constituents of EOs depend on the plant species, climatic conditions, and place of origin. EOs contain 85–99% of low molecular weight volatile and 1–15% of non-volatile components. The volatile constituents are a mixture of odoriferous compounds, such as terpenes, terpenoids, and other aromatic and aliphatic constituents. These volatile compounds take various chemical forms, including alcohols, acids, esters, aldehydes, ketones, amines, amides, sulfides, phenols, oxides, and heterocycles, but are mainly terpenes, terpenoids, and aromatic phenols, which play major roles in the composition of EOs [19]. Terpenes are a class of natural products found in EOs, constituting hydrocarbons with H and C atoms arranged in chains. These hydrocarbons can be arranged into acyclic, alicyclic (monocyclic, bicyclic, and tricyclic), or aromatic forms. Terpenes are made from naturally occurring hydrocarbons derived from a combination of isoprene units with a molecular formula of (C_5_H_8_)n and synthesized in the cytoplasm of plant cells via the mevalonic pathways [20]. The main terpenes are monoterpenes (C_10_H_16_) and sesquiterpenes (C_15_H_24_), which constitute 90% of EOs, but longer chains such as diterpenes (C_20_H_32_), sesterterpenes (C_25_H_52_), and triterpenes (C_30_H_54_) also exist at low concentrations. Examples of terpenes include pinene, *p*-cymene, terpinene, camphene, limonene, and sabinene. Terpenoids are aromatic, aliphatic acid esters and phenolic compounds, and a modified class of terpenes containing oxygen with different functional groups. Terpenoids undergo biochemical modifications via enzymes, wherein some oxidized methyl groups often remove or replace oxygen atoms [21]. Terpenoids are further subdivided into alcohols, aldehydes, ethers, esters, ketones, phenols, and epoxides. Carvacrol, menthol, geraniol, thymol, linalool, linalyl acetate, citronellal, and piperitone are good examples of terpenoids. The aromatic compounds derived from phenylpropane occur less frequently than terpenes. Aromatic compounds include aldehydes (cinnamaldehyde), alcohols (cinnamic alcohol), phenols (eugenol and chavicol), methoxyderivatives (estragole, anethole, and elemicin), and methylenedioxy compounds (apiole, myristicin, and safrole) [22]. The chemical structures of the major constituents of EOs are depicted in Figure 1 and Table 1.

## 3. Extraction Methods of Essential Oils

EOs are extracted by pharmaceutical industries, through either conventional/classical methods or advanced/modern technologies, from different raw plant materials. Extraction is normally dependent on the state and form of the botanical material used to determine the quality of EOs. Hydrodistillation, solvent extraction, and maceration methods have been traditionally used; however, these methods have some disadvantages such as poor efficiency, longer extraction time, loss of volatile compounds, degradation of unsaturated compounds, and toxic solvent residues [31]. Alternative and advanced techniques such as supercritical fluid (SCF), microwave-assisted extraction, and ultrasound-assisted extraction (UAE) methods have been developed to overcome the above problems.

### 3.1. Conventional Extraction Methods

#### 3.1.1. Hydrodistillation

Hydrodistillation is the simplest and oldest method of oil extraction, which includes a heating source, vessel, condenser, and a decanter to collect the condensate. The process involves complete immersion of the material directly into boiling water. The oil released from the oil glands/ducts/cells in the plant tissue due to the influence of boiling water and steam, and indirect cooling with water condenses the vapor mixture of water and oil. The main advantage of this technique is that the required material is distilled at a temperature less than 100 °C. Some important EOs, such as rosemary, geranium, oregano, lemon, lavender, sage cumin, clove, thyme, basil, and garden mint are extracted using this method [32]. There are three types of hydrodistillation for extracting EOs: water distillation, water and steam distillation, and direct steam distillation [33]. Hydro-diffusion, hydrolysis, and decomposition by heat are the three main physicochemical processes involved in the hydrodistillation process. Regarding water distillation, the material is immersed in warm water or placed on a holed plate above the bottom of the still, and then the mixture is subjected to distillation [34]. Water and steam distillation is similar to water distillation, but steam is generated either in a boiler or within the still until it starts volatilizing EOs in the biomass. In direct steam distillation, the process is carried out by passing dry steam through the material and allowing for sufficient contact time, and then mixture of hot vapors is collected and condensed [35].

#### 3.1.2. Solvent Extraction

In this method solvents such as acetone, hexane, methanol, ethanol, and petroleum ether are used to extract EOs from fragile or delicate flower materials such as jasmine, narcissus, hyacinth, and tuberose, which cannot be extracted using heat or steam distillation. In this process, the materials are treated with solvent and the mixture is mildly heated, followed by filtration and evaporation of the solvents. The filtrate or concentrate contains resin (resinoid) or concrete (mixture of wax, fragrance, and EO). The concrete substance is mixed with pure alcohol and distilled at a low temperature to dissolve the EO. During the distillation process, the alcohol absorbs the fragrance and is evaporated, leaving the aromatic oil residue. However, this method requires more time to process, thus making the oil obtained more expensive than that obtained via other methods. Some EOs, including lemon, sage, chasteberry, and those from the Apiaceae family, are extracted using this method [32].

#### 3.1.3. Maceration

Maceration is a simple extraction technique that is conducted at room temperature for a minimum of 3 days with frequent agitation. In this process, the coarsely ground powder material is placed in a closed vessel with a suitable solvent (such as water, alcohol, or oil) and allowed to stand with frequent agitation until the soluble matter is dissolved. This technique is used for the extraction of thermolabile compounds, which require maceration in warm water to release their EOs. Wintergreen leaves, brown mustard, garlic, bitter almonds [31], chokeberry, and strawberry [36] EOs are extracted using this technique. The main disadvantages of this method are that it requires a long extraction time and has low extraction efficiency.

### 3.2. Alternative Extraction Methods

#### 3.2.1. Supercritical Fluid Extraction

Supercritical fluid extraction is a well-documented and efficient alternative to obtain pure perfumes, fragrances, and active ingredients with no traces of solvents compared to conventional extraction techniques. Carbon dioxide (CO_2_) is the most commonly used SCF owing to its modest critical parameters, such as low cost, non-toxicity, easy availability, facile recovery from the product, and critical temperature and pressure. The plant materials are placed inside an extraction vessel in which the SCF is loaded from the bottom under a specific flow rate through a depressurization valve until the extraction conditions are reached. Eventually, all the extracts will pass over into the collector and into an analytical instrument to measure the extraction yield. The SCF state is primarily determined by two factors: the critical pressure and the critical temperature of the fluid. SCF has a relatively lower viscosity, and higher diffusivity can permit better transport properties than liquids and high density combined with solvent power, allowing for selective extractions [37]. The SCF extraction method is used to extract important EOs such as thyme, rosemary, cumin seed, fennel, anise, oregano, coriander, clove, lemon, and marjoram. [32].

#### 3.2.2. Microwave-Assisted Extraction

Microwaves have attracted much attention in extraction technologies owing to their unique heating mechanism, higher yield, shorter extraction time, and low cost under atmospheric conditions. However, this method requires a larger quantity of organic solvent and is not considered environment friendly compared to the SCF method. In this method, materials are immersed in a solvent and exposed to microwave energy. As the materials are heated up, high pressure is generated within the cell walls of the materials. The high-pressure build-up and severe thermal stress within the glands cause swelling, stretching, and rupture of the cell walls, which facilitates the release of constituents. A solvent-free microwave extraction method was also developed by Chemat et al. [38] based on a relatively simple and similar principle with a combination of heating energy and dry distillation, without the use of any solvents. Many EOs such as those of pepper, coriander, camphor, basil, epazote, lemon, rosemary, garden mint, and thyme are extracted at a laboratory scale using this technique.

#### 3.2.3. Ultrasound-Assisted Extraction

Ultrasound-assisted extraction (UAE) is a simple, efficient, and inexpensive method that uses ultrasonic wave energy for extraction. UAE is an effective, clean process because it allows the intensification and selectivity of EOs by stimulating their release from plant materials. Additionally, ultrasound increases the penetration of the solvent into the cell wall of plants through cavitation, which in turn facilitates the release of intracellular products from the plant. The extraction mechanism involves cavitation by the solvent, heat transfer through the cell walls, and breakdown of microscopic bubbles. The cavitation effect is due to the passage of ultrasonic waves, which can lead to cell destruction and improve the extraction rate of EOs in a shorter time. Several parameters, such as the influence of temperature, ultrasonic frequency, time, and type of solvent significantly affect the yield of EOs. UAE is considered one of the easiest, fastest, and more suitable methods for the extraction of thermolabile, unstable, and other natural products [39]. Jatropha, caraway, clove, sweet wormwood, eucalyptus, sage, rosemary, thymus, pomegranate, garlic, and grape are some of the EOs extracted using UAE. Extraction of EOs can be carried out by various means as shown in Table 2.

## 4. Antioxidant Activity of Essential Oils

Antioxidants neutralize unstable substances called free radicals developed in the form of reactive oxygen species (ROS), which are responsible for oxidative stress, DNA damage, and damage to cell membranes and other parts of the cell. Overproduction of ROS leads to many diseases such as Parkinson’s disease, Alzheimer’s disease, cardiovascular diseases, multiple sclerosis, cognitive impairment, cancer, and cardiac failure. Oxidative stress is a phenomenon caused by the interaction, production, and accumulation of oxidative free radicals and associated ROS, such as superoxide anions, hydroxyl radicals, and hydrogen peroxide [51]. Several studies have revealed that EOs are natural sources of antioxidants with various modes of action, such as free radical scavenging, prevention of chain initiation, reducing agents, termination of peroxides, quenching of singlet oxygen, and binding of metal ion catalysts [32]. In recent years, the food industry has sought natural and low-cost antioxidants from plant sources to substitute for synthetic antioxidants such as butylated hydroxyl toluene, butylated hydroxyl anisole, propyl gallate, and tert-butyl hydroquinone because of their negative health consequences.

The majority of natural antioxidants are phenolic and terpenolic compounds with the most important groups being flavonoids, tocopherols, and phenolic acids. The hydroxyl group of phenolics is directly attached to the carbon atom of the aromatic ring and the hydrogen atom donated to free radicals, thereby preventing the oxidation of other compounds [52]. Recently, numerous studies have investigated the antioxidant properties of different EOs, evaluating parameters such as total phenol, flavonoid, flavonol, catechin, phenolic acid, lignan, and tannin contents. Among the main constituents of EOs, terpenoids, sulfur-containing components, and phenolic compounds such as carvacrol, thymol, eugenol, and linalool, possess the highest scavenging activity of EOs [53]. In addition, several nonvolatile components such as rosmarinic acid, caffeic acid, carnosol, and quercetin have special radical chemistry that allows them to express potential antioxidant activity [54].

Many in vitro experiments involving 2,2-diphenyl-1-picrylhydrazyl free radical (DPPH), oxygen radical absorbance capacity, ferric reducing antioxidant power (FRAP), 2,2′-azino-bis-3-ethylbenzothiazoline-6-sulfonic acid (ABTS), reducing power, total phenolic content (TPC), and total flavonoid content (TFC) have been used to evaluate the antioxidant effects of different EOs. In addition, cupric ion reducing antioxidant capacity, Trolox equivalent antioxidant capacity, *β*-carotene-linoleic acid bleaching (BCB), ferrous ion chelating (FIC), thiobarbituric acid reactive substance (TBARS), and phosphomolybdate assays have been used to evaluate the antioxidant activity of EOs. However, differences in these methods may lead to difficulties in meeting protocol-specified procedures, and thus, improvement and modification of these methods continues to provide the most reliable techniques. Numerous studies have highlighted the antioxidant activities of EOs, including cinnamon, coriander, eucalyptus, clove, cumin, juniper, basil, thyme, and rosemary. Several studies have reported the antioxidant properties of different EOs with their main chemical compositions and in vitro assays (Summarized in Supplementary Table S1).

## 5. Mode of Antioxidant Action of Essential Oils

**Figure 2:**
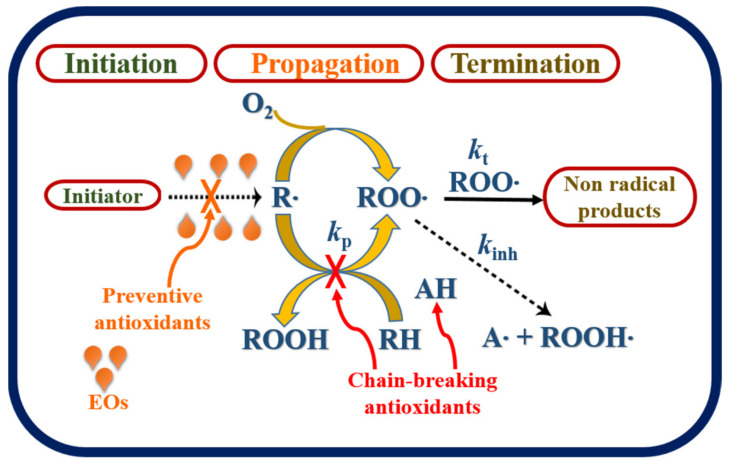
Proposed mechanism of hydrocarbon autoxidation and antioxidant properties of EOs. The typical three phases of free radical chain reactions, initiation, propagation, and termination are shown. RH represents a hydrocarbon, R∙ is an alkyl radical produced by the removal of a hydrogen atom from RH, ROO**∙** is the peroxy radical formed by the reaction of R∙ with molecular oxygen; k_p_ and k_t_ are the propagation and termination rate constants, respectively, and k_inh_ is the inhibition rate constant.

Autoxidation is a free radical chain reaction mechanism that involves three steps: initiation, propagation, and termination. Antioxidants are compounds capable of donating hydrogen radicals (H**∙**) to free radicals to prevent oxidative damage and stop the propagation reaction, which is called a chain reaction. In the initiation stage of the chain reaction, a hydrogen atom (H**∙**) is distracted from a neighboring carbon to a double bond in an unsaturated fatty acid substrate (RH), forming the alkyl R**∙** radical (free radicals) to initiate the autoxidation chains. This alkyl radical can react at a diffusion-controlled rate with molecular oxygen and create peroxyl (ROO**∙**) radicals that parallel the autoxidation of hydrocarbons. Cyclically, these radicals may be stable in the following propagation stage by attacking another susceptible molecule of the substrate (RH) to yield a lipid hydroperoxide (ROOH), the oxidized substrate and a new radical (R**∙**) [15]. During the termination stage, the chain reaction continues for many cycles until two radical species quench or react with each other to form non-radical species or stable products [55]. The probable mechanism and expected reaction steps of hydrocarbon autoxidation and antioxidant protection of EOs are illustrated in Figure 2.

The antioxidant activity of EOs depends on the presence of bioactive components that can quench peroxyl radicals or inhibit the oxidation reaction of the organic materials. The most relevant components such as phenolic moieties (thymol, eugenol, and carvacrol), terpenoids with a cyclohexadienyl structure (*γ*-terpinene and menthe-1,4-dien-7-al), carotenoids (*β*-carotene and xanthophyll), and vitamins E and C have demonstrated higher antioxidant capacity [56]. Phenolic components directly scavenge reactive species by providing hydrogen atoms or by electron donation from hydroxyl, peroxyl, and superoxide groups, acting as chain-breaking antioxidants [57]. These compounds may suppress lipid peroxidation and recycling other antioxidants, such as *α*-tocopherol which is the most potent member of this family. The antioxidant mechanism of carotenoids is related to their higher electron-donating capacity. *α*-Tocopherol produces a semi-stable lipid hydroperoxide by donating a hydrogen atom to a peroxy lipid radical and effectively blocking lipid peroxidation [58]. The antioxidant mechanism of vitamin C is based on hydrogen atom transfer to peroxy radicals, quenching of singlet oxygen, and removal of molecular oxygen. Vitamin E is also a potent radical-scavenging antioxidant that inhibits lipid peroxidation and scavenges to break chain propagation efficiently.

## 6. Antibacterial Activity of Essential Oils

In recent years, a large number of essential oils and their chemical constituents have been investigated for their antibacterial activities against many food-borne pathogens, including gram-positive, gram-negative, and spoilage microorganisms. The antibacterial efficacy of EOs differs in different plants and different target bacteria depending on their structure. Some chemical constituents of EOs are dominated by a range of compounds, such as phenolics, aldehydes, ketones, terpenoids, ethers, and epoxides, which exhibit inhibitory activities against various bacterial pathogens. The association of some major constituents of EOs including thymol, carvacrol, eugenol, cinnamic aldehyde, and *p*-cymene, which easily penetrate the lipids of the cell membrane, disrupt the cell membrane, cause loss of membrane integrity and cellular contents, and finally lead to cell death [59]. Antibacterial screening of different EOs is usually conducted using well-known and common in vitro techniques such as agar disk diffusion, agar dilution, time-kill, and checkerboard methods. The bactericidal activity was estimated by measuring the minimum inhibitory concentration (MIC) and minimum bactericidal concentration (MBC). MIC is defined as the lowest concentration of antibacterial agent that completely inhibits the visible growth of the microorganism in micro-dilution wells or tubes. MBC is the lowest concentration of an antibacterial agent required to prevent or kill 99.9% of a particular bacterium over a fixed or somewhat extended period under specific conditions [60].

Despite their thicker peptidoglycan layer, gram-positive bacteria are more receptive to certain cell wall targeting EOs than gram-negative bacteria, due to the absence of the outer membrane. Generally, thyme, oregano, clove, tea-tree, lemon grass, cinnamon, bay, and rosewood oils are the most active antimicrobials. Terpenoids such as menthol, thymol, and linalool present in EOs with oxygen atoms or methyl groups show greater antibacterial activity. Terpinene, limonene, and 1,8-cineole, with highly active functional groups, show enhanced antibacterial activities [61]. The carvacrol precursor cymene, a monoterpene with a benzene ring, shows the highest affinity for microbial membranes and perturbs the potential of the cell membrane. However, the antimicrobial activity of the non-phenolic compound limonene is influenced by the alkyl group [62]. The antibacterial efficacy depends on different characteristics of compounds, such as chemical groups, ring structures, and side chains of EOs, and their components [63]. Several reports have described the interactions between the chemical constituents and antimicrobial activities of EOs. Similarly, the antimicrobial activities of EOs are closely related to their major and minor chemical constituent interactions [64]. The antibacterial properties of some commonly reported EOs with their major constituents are listed in Supplementary Table S2.

## 7. The Mode of Antibacterial Action of Essential Oils

**Figure 3:**
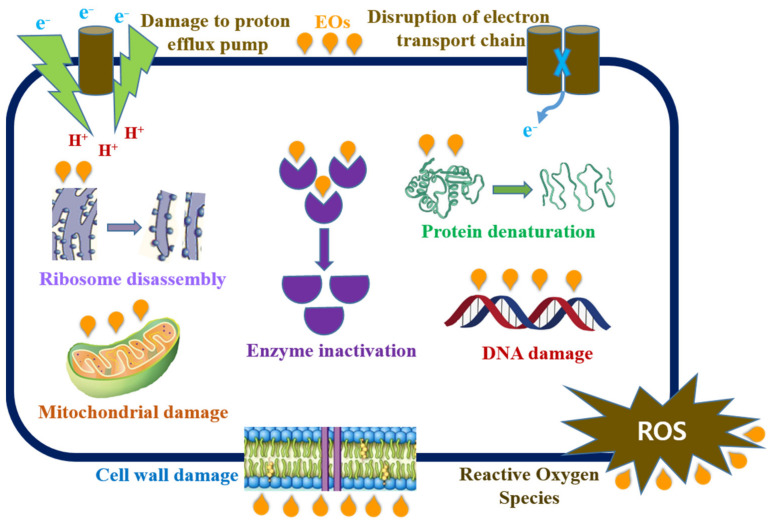
Proposed mechanism of antibacterial action of essential oils.

The antibacterial mechanisms of EOs depend mainly on their respective structures, chemical constituents, functional groups, and their synergistic interactions. Overall, the mechanism of antibacterial action is mediated by a series of biochemical reactions in the cell and the type of chemical constituents. Moreover, the mode of action differs for various types of microorganisms, such as gram-positive and gram-negative bacteria, which are related to the structure and outer membrane composition of cell walls. Generally, gram-negative bacteria exhibit natural resistance or are less susceptible to a variety of EOs because of their hydrophilic lipopolysaccharide outer membrane, which creates a barrier or limits the diffusion of macromolecules and hydrophobic compounds [20]. However, gram-positive bacteria may facilitate the percolation of hydrophobic compounds due to the presence of lipoproteins in the outer membrane of the cell [65]. The antibacterial action of EOs causes structural and functional damage to the cell membrane and increases membrane permeability. This can be directly associated with the loss of inorganic ions, membrane potential reduction, nucleic acid synthesis, protein dysfunction, proton pump collapse, and depletion of ATP [66]. Most of the bioactive components present in EOs have several targets; therefore, it is difficult to predict the susceptibility of various strains with different EOs. The components of EOs attach to the cell surface and thereafter penetrate the phospholipid bilayer of the cell membrane [12]. This may lead to the plasma membrane being more permeable to ions and protons, coagulating in the cytoplasm, damaging lipid and protein layers, leakage of intracellular ingredients, disrupting enzyme systems, affecting cellular metabolism, and consequently leading to cell death. A possible mode of the antibacterial action of EOs is illustrated in Figure 3.

The phenolic contents of EOs, such as carvacrol, thymol, eugenol, and oregano have potent antibacterial activities against both gram-positive and gram-negative pathogens. Interestingly, both the hydrophilic and hydrophobic parts of the phenolic compounds facilitate antibacterial activity. Specifically, the hydrophilic part interacts with the polar part and the hydrophobic part with the inner part of the bacterial cell membrane [67]. Analysis of the underlying mechanism of cinnamon EO showed that the addition of cinnamon EO destroyed the cell membrane at the MIC level, whereas it killed *E. coli* and *S. aureus* cells at the MBC level [68]. Similarly, the *E. coli* cell membrane shrinks, breaks, and leads to the leakage of electrolytes, proteins, ATP, and DNA materials, which results in disorder, decomposition, and death of cells when treated with black pepper EO [69]. Another study described the damaged cell membrane of *E. coli* and *L. monocytogenes* by increased leakage of K^+^ ions, resulting in loss of integrity and increased permeability when treated with Forsythia EO [70]. Similarly, treatment with *Litsea cubeba* EO ruptured the cells of *E. coli*, resulting in oil penetration and the destruction of the outer and inner membranes of the cells [71]. The mode of antibacterial action varies with the type of EO and its constituents, targeted site, interactions with the surrounding environment, and strain of the pathogens used.

## 8. Role of Essential Oils in Active Food Packaging Applications

EOs are widely used as natural additives to prolong the shelf-life of food and ensure food safety and quality in a sustainable manner. Nowadays, the demand for smart or active packaging in the food processing industry is growing mainly due to the extension of the shelf life of packaged food products and the prevention of food from spoiling. Many investigations have been conducted using either pure Eos or formulations of Eos in different storage containers, such as tin, glass, cardboard, and polyethylene, and significant improvements have been observed in their shelf lives. The major components of Eos are potential antimicrobials and antioxidants applied in most common types of food such as fruits, vegetables, meat, fish, dairy products, bread, and bakery foods [72]. Eos may quickly decompose or degrade because of their unstable volatile constituents when applied directly to the food matrix. The stability of Eos depends on many extrinsic and intrinsic factors that lead to chemical reactions. The extrinsic factors include the presence of oxygen, exposure to light, temperature, and humidity, whereas the intrinsic factors include the chemical structure and impurities present in Eos [73].

Thus, several promising approaches have been introduced to improve and enhance the stability of Eos by encapsulating them with polymers, liposomes, and solid lipid nanoparticles. Generally, the selected packaging (biopolymer) film matrix incorporated with Eos is prepared by the casting method, in which films are dispersed in casting solution followed by evaporation of the solvent [74]. Analyses of the optical, mechanical, and barrier properties of the active films are essential to aid their choice for use in different food matrices. Analysis of the mechanical properties, including tensile strength, breaking strength, and elastic modulus of the polymer film matrix is essential. Similarly, analysis of the chemical and physical properties, such as solubility, thickness, water activity, thermogravimetry, and permeability of water vapor, oxygen, and carbon dioxide, in the film matrix is required to ensure the quality and safety of food [74]. Cinnamon, thyme, oregano, jasmine, rosemary, cumin, peppermint, tea tree, clove, eucalyptus, geranium, lemon, mandarin, rosewood, lavender, lemongrass, and palmarosa are the most commonly used EOs, together with their constituents, in packaging systems.

Several authors have reported the potential of EOs to improve the quality and safety of food and extend the shelf life of such products [75]. The best results were achieved when oregano and clove EOs were incorporated into cassava bagasse-polyvinyl alcohol (PVA), resulting in the inhibition of, or reduction in, total microbial viability, including molds, yeasts, and both gram-positive and gram-negative bacteria [76]. In another study, thyme EO encapsulated with curdlan-PVA showed improvement in antioxidant activity and extension of the shelf life of chilled meat [77]. Similarly, rosemary EO encapsulated with whey protein isolate/cellulose nanofiber film increased the shelf life of lamb meat for up to 15 days when compared to the control meat after 6 days [78]. Citral and eugenol EOs with sodium alginate are capable of protecting against microbial growth and improving the postharvest quality of strawberry fruits [79]. Similar results were observed with citronella, lemongrass, and basil EOs, which could significantly control crown rot, anthracnose, and increased banana shelf life with texture and flavor [80].

In fact, foods are more susceptible to oxidation; thus, the most common cause of food spoilage is oxidative rancidity and microbial growth. However, packaging films loaded with different EOs showed a better antioxidant capacity for DPPH, FRAP, and ABTS assays. For instance, edible pectin film with cinnamon EO increased the antioxidant capacity and reduced the bacterial growth of fresh-cut peaches [81]. The shelf life of fresh Mediterranean swordfish increased for up to 13 days when treated with thyme EO with low-density polyethylene [82]. Similarly, chitosan/montmorillonite incorporated with rosemary EO extended the shelf life of poultry meat for 15 days [83]. The higher antioxidant effects of oregano EO incorporated with soy protein film resulted in ground beef patties that control primary lipid oxidation and lipid hydrolysis [84]. Thyme is one of the most potent antioxidants, followed by rosemary, which could be due to their chemotypes and a high percentage of terpenes. Hence, EOs should be considered as potential antioxidants based on the evidence presented in these reports.

## 9. Synergistic Advantages of Different Essential Oils

Multidrug-resistant bacteria are considered one of the most significant emerging threats to human health worldwide. Therefore, there is an urgent need to find alternative strategies to prevent and treat bacterial infections resulting from MDR bacteria with new structures and novel mechanisms. A combination or interaction between different EOs and their major or minor constituents may lead to additive, synergistic, or antagonistic effects [14]. An additive effect is generally considered as the combined effect of two or more compounds being equal to the sum of individual effects. Synergy is defined as the combined effect of two or more compounds that is greater than the sum of the individual effects. An antagonistic effect is observed when the effects of two or more substances in combination are lesser than the sum of the individual effects of those substances. Synergy is a pillar of modern pharmacology and medicine, because many diseases require treatment that consists of a mixture or combination of various drugs or antimicrobials taken at once [85]. Synergistic interaction enhances the antimicrobial and antioxidant activities by employing the combination of two or more EOs in the best possible manner, thereby reducing the required doses of the combined agents. This potentially benefits patients for the treatment of the disease, while minimizing side effects and adverse reactions [86].

The antioxidant and antibacterial activities of different EOs may depend on one or two of the major constituents of the oil. Compared to the use of a single EO or compound, the combination of two or more EOs or their constituents can improve their antimicrobial activities, preservative effects, and reduce organoleptic impact in food even at lower doses. A mixture of two or more EOs can increase the diversity of components and result in multiple sites of action. There has been an increasing demand for synergistic effects of EOs and their constituents due to their multiple biochemical processes and interactive antibacterial effects in food preservation [87]. Interestingly, combinations of phenylpropanoid (eugenol and chavicol) and phenolic monoterpenes (thymol and carvacrol) with other components were found to increase bioactivities, including antimicrobial, antioxidant, antiherbivore, and other pharmaceutical activities. For instance, the combination of phenolics with monoterpene alcohols exhibited the highest synergistic effect against *E. coli* pathogens [87]. Another study revealed that the binary combination of carvacrol and thymol and the ternary combination of carvacrol, thymol, and eugenol had the most synergistic effect against *L. innocua* [88].

Antioxidant and antibacterial evaluation of different herbs and spices such as cumin, coriander, black pepper, garlic, ginger, onion, turmeric, bay leaf, and mustard in combination showed that only coriander and cumin seed oils produced synergistic interactions, while others showed only additive effects [89]. This indicates that the proton-donating capability of coriander and cumin seed combinations was higher at low concentrations than other combinations. Similarly, the synergistic effect of oregano/thyme, cinnamon/thyme, mint/tea tree, and oregano/mint EOs in combination exhibited the highest antimicrobial activity because pathogens cannot develop resistance to multiple components of two or more EOs. Thus, the antioxidant and antibacterial potential can be increased by the synergistic interactions between different EOs or constituents of two or more extracts in combination. The synergistic antioxidant and antibacterial activities of some EOs combined with other EOs or constituents are summarized in Supplementary Tables S3 and S4, respectively.

It was revealed that the combination of different EOs produced synergism, resulting from the combined activities of several chemical constituents of EOs, and pathogens cannot easily develop resistance to multiple components of two or more EOs. Thus, synergistic interactions were observed between different EO combinations showing enhanced antioxidant and antibacterial activities with a reduction in the required doses of the combined agents.

The oregano EO (rich in thymol and carvacrol) was the most used EO combined with cinnamon, rosemary and thyme for many industrial applications. Similarly, thyme and clove EOs (rich in thymol and eugenol respectively) combined with cumin and cinnamon attributed synergism effects (Tables S3 and S4). The multicomponent nature and complexity of their structure in combination may work synergistically and improve the bioavailability of the combined agents, affecting multiple biochemical processes in the bacteria. The synergistic interaction of different EOs and their components as antioxidants and antimicrobial agents can prevent food spoilage caused by oxidation and microbial action, thereby increasing consumers’ acceptance for packed food materials.

## 10. Concluding Remarks and Prospects for Future Research

The problem of increasing MDR pathogens is a growing health threat and a great challenge for researchers, clinicians, and pharmacological industries to develop or discover new and alternative antimicrobial agents. Similarly, the food safety challenges in modern food supply systems must be overcome to assure consumers of a safe, wholesome, nutritious, and antimicrobial approach to food packaging and safety management. EOs have been used as natural preservatives, additives, and antimicrobial agents in the food industry for many years to ensure the safety and quality of food products. Natural antimicrobials can replace existing preservatives, such as synthetic antioxidants and commercial antibiotics, because of their high cost, low effectiveness, and the organoleptic quality deterioration of the products. Hence, EOs and their constituents can lower the organoleptic quality deterioration of food products because of their low price, bioavailability, and wide range of biological activities with synergistic interactions.

Numerous studies have been conducted on the synergistic interactions of different EOs, including EOs/antibiotics, EOs/EOs, EOs/EO constituents, EOs/plant extracts, plant extract/plant extracts, phytochemical/antibiotics, and EO constituent/EO constituents. In addition, many researchers have reported EOs encapsulated with different metal and metal oxide nanoparticles to evaluate their potential antimicrobial activities. However, very few studies have been conducted on the synergistic effects of more than three EOs or their constituents. Further research is needed to explore the synergistic interactions of more than two or three EOs or constituents to determine the exact contributions of synergy among different EOs and their constituents. The exact mechanism of the antibacterial action of individual EOs and multiple EOs with their synergistic interactions is still lacking. Thus, the mode of action of individual and multiple EOs should be explored by systemic investigations of synergy among different constituents.

The present review highlights the information on EO extraction, composition, antioxidant and antibacterial properties, and shelf-life extension of packaged food. However, owing to the limited number of studies on the synergistic interaction of EOs, further research should focus on the effect of intrinsic and extrinsic parameters on the combinations of EOs. In addition, the molecular interactions of biopolymers with food matrices before formulating EOs in food to minimize organoleptic effects should be investigated. Furthermore, standard methods and possible toxicity evaluations of combined EOs and their components should be developed. The synergistic effects of EOs in combination or encapsulation could potentially reduce the organoleptic impact and simultaneously maximize their biological efficacy even at smaller doses. The comprehensive use of these modern and alternative preservative techniques can significantly extend the shelf life and fulfill consumer demands of taste, aroma, color, and texture of packaged food. Therefore, it can serve as a convenient packaging solution, which effectively increases the safety and shelf life of food products.

## Figures and Tables

**Table 1:** Major active chemical constituents of some important essential oils with molecular structure and plant sources.

Essential Oil Constituents	Examples	Chemical Formula	Molecular Structure	Plant Source	Reference
Terpenes	*α*-Pinene	C_10_H_16_	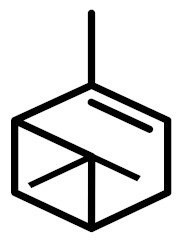	*Piper nigrum*	[23]
	Limonene	C_10_H_16_	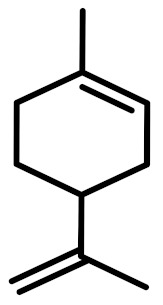	*Aloysia triphylla*	[24]
	Sabinene	C_10_H_16_	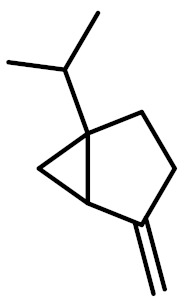	*Oenanthe crocata*	[25]
	*p*-Cymene	C_10_H_14_	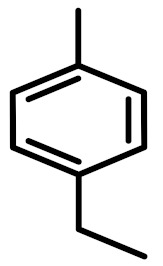	*Thymus fontanesii*	[26]
Terpenoids	Linalool	C_10_H_18_O	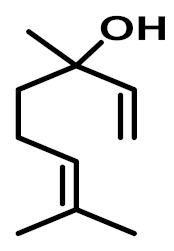	*Citrus limon*	[27]
	Thymol	C_10_H_14_O	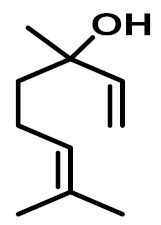	*Thymus fontanesii*	[26]
	Menthol	C_10_H_20_O	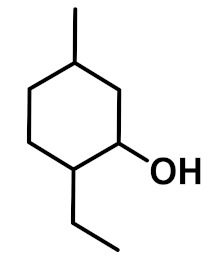	*Aetheroleum menthae piperitae*	[28]
	Carvacrol	C_10_H_14_O	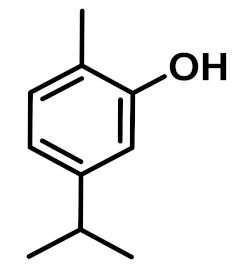	*Zataria multiflora*	[29]
Aldehydes	Cinnamaldehyde	C_9_H_8_O	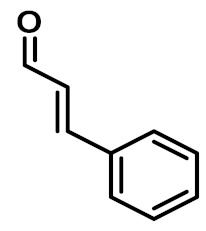	*Cinnamomum zeylanicum*	[23]
Phenols	Eugenol	C_10_H_12_O_2_	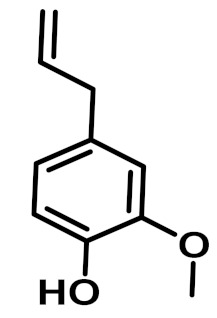	*Syzygium aromaticum*	[23]
	Chavicol	C_9_H_10_O	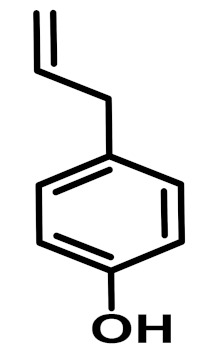	*Ocimum ciliatum*	[30]

**Table 2:** Extraction of essential oils from various sources using different methods.

Essential Oils	Plant Source	Major Components	Extraction Methods	Ref.
Rosemary	*Rosmarinus officinalis*	Cineole, camphor, α-pinene	Hydrodistillation	[40]
Neroli	*Citrus aurantium*	Linalyl acetate, linalool, α-terpineol	Hydrodistillation	[41]
Tobacco	*Nicotiana tabacum*	β-Damascenone, β-damascone, cembrene	Solvent extraction	[42]
Lemon	*Citrus latifolia*	Limonene, γ-terpinene, β-pinene	Solvent extraction	[43]
Chokeberry	*Aronia melanocarpa*	Cyanidin-3-galactoside, quercetin-3-glucoside	Maceration	[44]
Thyme	*Thymus serpyllum*	Rosmarinic acid, salvianolic acid	Maceration	[45]
Lavender	*Lavandula angustifolia*	Linalool, camphor, terpinen-4-ol	Supercritical fluid extraction	[46]
Mint	*Mentha spicata*	Menthone, carvone, limonene	Supercritical fluid extraction	[47]
Basil	*Ocimum basilicum*	Linalool, methyl-cinnamate, β-cubebene	Microwave-assisted extraction	[48]
Epazote	*Chenopodium ambrosioides*	Ascaridole, carvacrol, caryophyllene oxide	Microwave-assisted extraction	[48]
Celery	*Apium graveolens*	Limonene, β-selinene, sedanenolid	Ultrasound-assisted extraction	[49]
Sweet wormwood	*Artemisia annua*	Camphor, eucalyptol, myrtenol	Ultrasound-assisted extraction	[50]

## Data Availability

Not applicable.

## Supplementary Materials

The following are available online at https://www.mdpi.com/article/10.3390/biom11091267/s1, Table S1: Overview of studies on antioxidant properties of selected essential oils, Table S2: Antibacterial efficacy of various essential oils and their components against different pathogenic bacteria, Table S3: Synergistic antioxidant effects of different essential oils, their major constituents, with various in vitro assays, Table S4: Combination of different essential oils with major constituents and their antibacterial interactions against various microorganisms.
