# Supplementary material for: Synergistic Antioxidant and Antibacterial Advantages of Essential Oils for Food Packaging Applications
Source: Biomolecules. 2021 Sep 2;11(9):1267. doi: 10.3390/biom11091267 (PMC8466708; doi:10.3390/biom11091267)
Supplement: Supplementary file 1 [file biomolecules-11-01267-s001.zip › biomolecules-1293093-supplementary.pdf]

Supplementary Materials

Review

Synergistic Antioxidant and Antibacterial Advantages of Essential Oils for Food Packaging Applications

Nagaraj Basavegowda and Kwang-Hyun Baek *

Department of Biotechnology, Yeungnam University, Gyeongsan 38451, Gyeongbuk, Korea; nagarajb2005@yahoo.co.in
* Correspondence: khbaek@ynu.ac.kr; Tel.: +82-52-810-3029

Table S1. Overview of studies on antioxidant properties of selected essential oils.

Essential Oils	Plant Source	Major Components	Molecular Structure	Assay	Ref.
Shady calamint	*Clinopodium umbrosum*	*β*-Caryophyllene, germacrene D, spathulenol	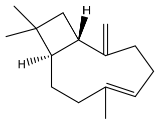	DPPH, FRAP, LPI, TAC	[1]
Lemon beebrush	*Aloysia triphylla*	Limonene, geranial, myrcene	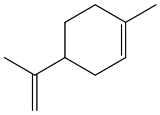	DPPH, TPC	[2]
Tipollo	*Minthostachys mollis*	Pulegone, menthone, limonene	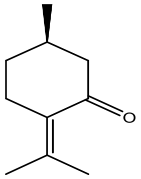	DPPH, TPC	[2]
Dittany	*Origanum dictamnus*	Carvacrol, *γ*-terpinene, *p*-cymene	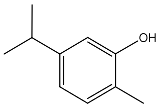	DPPH	[3]
Thyme	*Thymus fontanesii*	Thymol, *p*-cymene	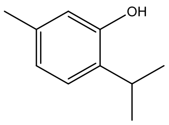	DPPH, FRAP	[4]
White Wormwood.	*Artemisia herba-alba*	Camphor, chrysanthenone	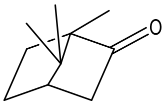	DPPH, FRAP	[4]
Rosemary	*Rosmarinus officinalis*	1,8-Cineole, camphene, *α*-pinene	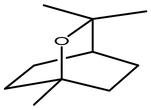	DPPH, FRAP	[4]

Cinnamon	*Cinnamomum zeylanicum*	Cinnamaldehyde, *β*-caryophyllene	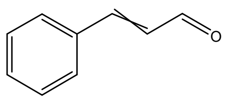	DPPH, BCB, FIC	[5]
Clove	*Syzygium aromaticum*	Eugenol, *β*-caryophyllene	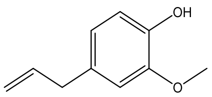	DPPH, BCB, FIC	[5]
Black pepper	*Piper nigrum*	*α*-Pinene, *β*-pinene, *β*-Caryophyllene	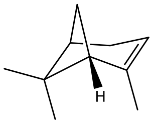	DPPH, BCB, FIC	[5]
Winter savory	*Satureja montana*	*p*-Cymene, borneol, *γ*-terpinene	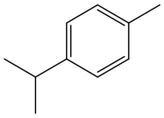	DPPH	[6]
Oregano	*Origanum vulgare*	Carvacrol, *γ*-terpinene, *p*-cymene	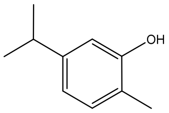	DPPH	[6]
Lemon	*Citrus limon*	*β*-Pinene, Limonene, Linalool	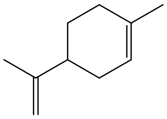	DPPH, BCB	[7]
Thyme	*Thymus quinquecostatus*	Thymol, *γ*-terpinene, *p*-cymene	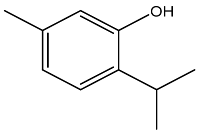	DPPH, ABTS, FRAP	[8]
Laminaria (Kumbu)	*Laminaria japonica*	Tetradecanoic acid, hexadecanoic	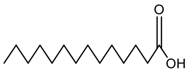	DPPH, ABTS	[9]
Spearmint	*Mentha viridis*	Carvone, 1,8-cineole, terpinen-4-ol	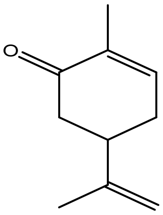	DPPH, ABTS, FRAP	[10]
Basil	*Ocimum basilicum*	Linalool, estragole, methyl cinnamate	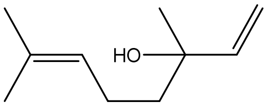	DPPH	[11]

Tea tree	*Melaleuca alternifolia*	Terpinen-4-ol, γ-terpinene, α-terpinene	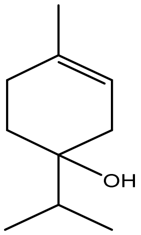	DPPH, TBARS	[12]
Neroli	*Citrus aurantium*	D-limonene, γ-terpinene, linalool	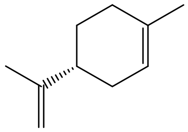	DPPH, ABTS	[13]
Turmeric	*Curcuma longa*	α-Turmerone, β-turmerone, ar- turmerone	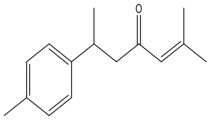	DPPH, ABTS	[14]
Costmary	*Tanacetum balsamita*	β-Thujone, α-thujone, eucalyptol	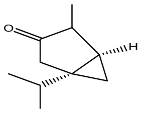	DPPH, FRAP	[15]
Tansy	*Tanacetum vulgare*	trans-Chrysanthenyl acetate, β-thujone, (E)-dihydrocarvone	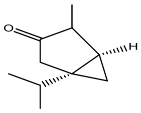	DPPH, FRAP	[15]
Shirazi thyme	*Zataria multiflora*	Carvacrol, thymol, thymol acetate	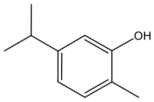	Phosphomolyb date assay	[16]
Ginger	*Zingiber officinale*	Camphene, β-phellandrene, 1,8-cineole	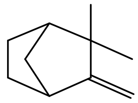	DPPH, FRAP, BCB	[17]
Peppermint	*Aetheroleum menthae piperitae*	Menthol, menthone, iso-menthone	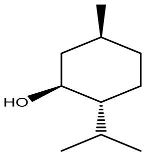	DPPH	[18]
Anise Hyssop	*Agastache foeniculum*	Methyl chavicole, limonene, spathulenol	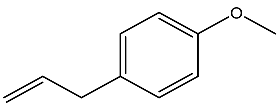	DPPH ABTS	[19]
Dill	*Anethum graveolens*	Neral, carvone, limonene	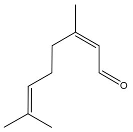	DPPH, FRAP, BCB	[17]

Terragon	*Artemisia dracunculus*	*p*-Allylanisole, ocimene (E)-*β*, ocimene (Z)-*β*	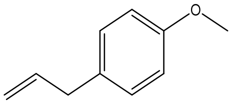	DPPH, TPC, TFC	[20]
Khat	*Catha edulis Forsk*	Limonene, tritetracontane, 1-phenyl-1,2-propanedione	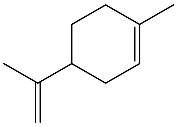	DPPH	[21]
Chamomile	*Ormenis mixta*	Germacrene, 1,8-cineole, cis-methyl isoeugenol	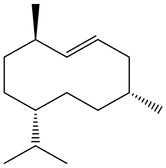	DPPH	[22]
Geranium	*Pelargonium asperum*	Citronellol, citronellyl formate, geraniol	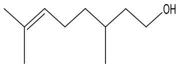	DPPH	[22]
Saxifrage	*Pimpinella saxifraga*	Anethole, pseudoisoeugenol, p-anisaldehyde	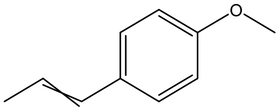	DPPH, FRAP	[23]
Pistachio	*Pistacia vera*	4-Carene, *α*-pinene, D-limonene	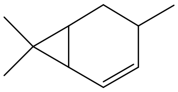	DPPH, FRAP	[24]
Jashir	*Prangos gaubae*	Germacrene D, caryophyllene oxide, (E)-caryophyllene	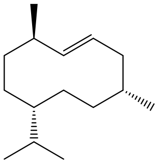	ABTS, FRAP	[25]
Cattley guava	*Psidium cattleianum*	*α*-Copaene, eucalyptol, *δ*-cadinene	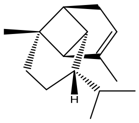	DPPH	[26]
Eucalyptus	*Eucalyptus globulus*	1,8-Cineole, *α*-pinene, o-cymene	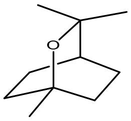	DPPH, BCB	[27]
Laurel	*Laurus nobilis*	1,8-Cineole, *α*-terpinyl acetate, 4-terpineol	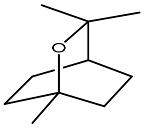	DPPH, FRAP, BCB	[17]

Table S2. Antibacterial efficacy of various essential oils and their components against different pathogenic bacteria.

Essential Oils	Plant Source	Major Constituents	Molecular Structure	Microbial Strains	Ref.
Lemon	*Citrus limon*	Limonene, β-pinene, α-pinene	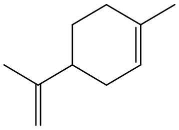	*S. aureus, E. faecalis, E. coli, K. pneumonia, P. aeruginosa*	[28]
Oregano	*Origanum vulgare*	Carvacrol, monoterpenoid phenol	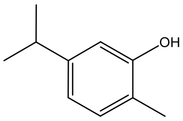	*S. aureus, E. faecalis, E. coli, K. pneumonia, P. aeruginosa*	[28]
Thyme	*Thymus vulgaris*	Thymol, γ-terpinene, carvacrol	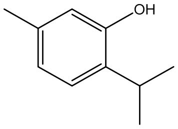	*S. aureus, E. faecalis, E. coli, K. pneumonia, P. aeruginosa*	[28]
Lavender	*Lavandula angustifolia*	Lonalool, linalyl acetate	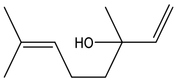	*S. aureus, Enterococcus sp.*	[29]
Peppermint	*Mentha spicata*	Menthol, mentone, cineol	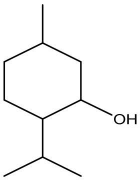	*S. aureus, E. coli*	[30]
Cajuput	*Melaleuca cajuputi*	1,8-Cineole, γ-terpinene, limonene	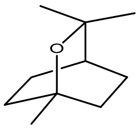	*S. aureus, S. epidermidis, B. cereus*	[30]
Cinnamon	*Cinnamomum Zeylanicum*	Cinnamaldehyde, eugenol	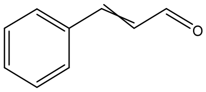	*S. aureus, E. coli, P. aeruginosa*	[31]
Clove	*Syzygium aromaticum*	Eugenol, β-caryophyllene	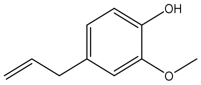	*S. aureus, E. coli, P. aeruginosa*	[31]
Eucalyptus	*Eucalyptus globulus*	1,8-Cineole, α-pinene	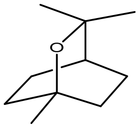	*S. aureus, E. coli*	[32]
Sage	*Salvia offcinalis*	Borneol, camphor, 1,8-cineole	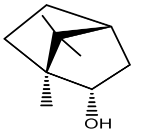	*B. subtillis, S. epidermidis*	[33]

Tea Tree	*Melaleuca alternifolia*	Terpine-4-ol, teripene, α-terpinene	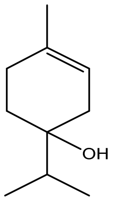	*S. aureus, E. coli, P. aeruginosa*	[34]
Caraway	*Carum carvi*	Limonene, carvone, β-myrcene	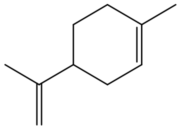	*E. coli, B. bronchiseptica*	[35]
Coriander	*Coriandrum sativum*	Linalool, λ-terpinene, α-pinene	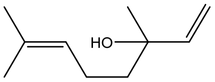	*E. coli, B. bronchiseptica*	[35]
Cumin	*Cuminum cyminum*	Cuminaldehyde, γ-terpinene,	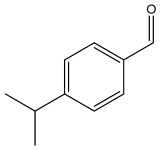	*E. coli, B. bronchiseptica*	[35]
Vassoura	*Baccharis dracunculifolia*	Spathulenol, trans-nerolidol	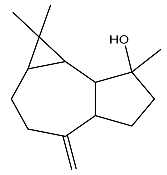	*S. aureus, B. cereus, P. aeruginosa*	[36]
Basil	*Ocimum basilicum*	Linalool, estragole	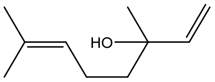	*S. aureus, S. pyogenes, E. coli*	[37]
Black pepper	*Piper nigrum*	β-Caryophyllene, caryophyllene oxide	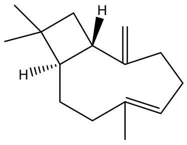	*B. cereus, L. monocytogenes, M. luteus*	[5]
Wintergreen	*Gaultheria procumbens*	Methyl salicylate, α-pinene, myrcene	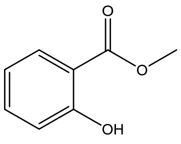	*P. syringae*	[6]
Armoise	*Artemisia herba alba*	β-Thujone, camphor, α-thujone	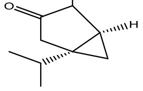	*S. aureus, E. coli, P. aeruginosa*	[38]
Dill weed	*Anethum graveolens*	Carvone, limonene, carvocrol	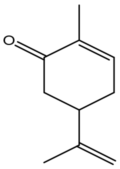	*S. aureus, E. coli*	[39]

Camphor	*Cinnamomum camphora*	D-camphor, linalool, cineole	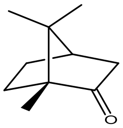	*B. subtillis, S. aureus, E. coli*	[40]
Lemon grass	*Cymbopogon citratus*	Citral α, citral β, nerol,	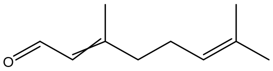	*S. aureus, E. coli*	[41]
Cardamom	*Elettaria cardamomum*	A-terpinyl acetate, 1,8-cineole, linalyl acetate	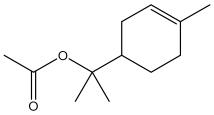	*S. aureus, E. coli*	[39]
Anise	*Pimpinella anisum*	Trans-anethole, p-anisaldehyde, methyl chavicol	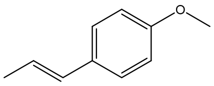	*B. subtillis, S. aureus, E. coli*	[40]
Rosemary	*Rosmarinus officinalis*	1,8-Cineole, camphor, α-pinene, limonene	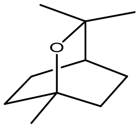	*B. subtillis, E. coli, L. monocytogenes*	[42]
Savory	*Satureja hortensis*	Thymol, γ-terpinene, *o*-cymene	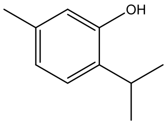	*E. coli, L. monocytogenes, S. aureus*	[43]
Pepper	*Zanthoxylum bungeanum*	Terpinene-4-ol, 1,8-cineole, limonene	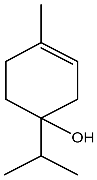	*B. subtillis, S. aureus, E. coli*	[40]
Zataria	*Zataria multiflora*	Thymol, *p*-cymene, carvacrol	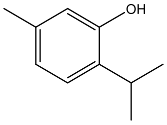	*B. cereus, S. aureus, E. coli*	[16]
Ginger	*Zingiber officinale Roscoe*	Zingiberene, β-phellandrene, sesquiphellandrene	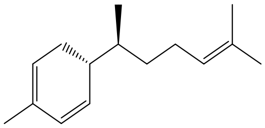	*E. coli, E. faecalis, K. pneumoniae*	[44]
Arina	*Psiadia arguta*	Isoeugenol, isoeugenyl acetate	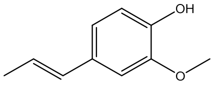	*S. epidermidis, S. aureus, E. faecalis*	[45]
Betel leaf	*Piper betle*	Eugenol acetate, eugenol, β-caryophyllene	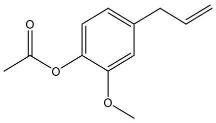	*S. epidermidis, S. aureus, E. faecalis*	[45]

Allspice	*Pimenta dioica*	Eugenol, methyl eugenol, *β*-caryophyllene	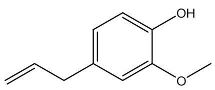	*S. epidermidis, S. aureus, E. faecalis*	[45]
Aniseed	*Pimpinella anisum*	Trans-anethole, estragole, *γ*-himachalene	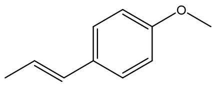	*C. perfringens*	[46]
Garlic	*Allium sativum*	Diallyl trisulfide, diallyl disulfide, methyl allyl trisulfide	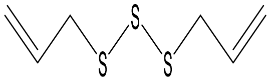	*E. coli*	[47]

Table S3. Synergistic antioxidant effects of different essential oils, their major constituents, with various in vitro assays.

Essential Oils	Plant Source	Major Constituents	Molecular Structure	Assay	Ref.
Thyme/ Rosemary	*Thymus fontanesii/ Rosmarinus officinalis*	Thymol, *p*-cymene/1,8-cineole, camphre	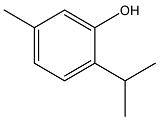	DPPH, FRAP	[4]
Thyme/White Wormwood.	*Thymus fontanesii/ Artemisia herba-alba*	Thymol, *p*-cymene/Camphor, chrysanthenone	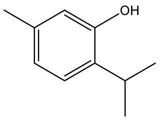	DPPH, FRAP	[48]
White Wormwood/Rose mary	*Artemisia herba-alba/ Rosmarinus officinalis*	Camphor, chrysanthenone/1,8-cineole, camphre	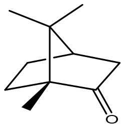	DPPH, FRAP	[48]
Coriander/ Cumin	*Coriandrum sativum/ Cuminum cyminum*	Linalool, *p*-coumaric acid/cuminaldehyde, *p*-cymene	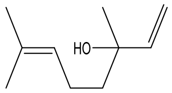	DPPH	[48]
Thyme/Celery/ Coriander	*Thymus vulgaris/ Apium graveolens/ Coriandrum sativum*	*α*-Pinene, thymol/citronellol, geraniol/linalool	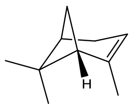	FRAP, ABTS	[49]
Thyme/Oregano	*Thymus leptobotrys/ Origanum compactum*	Carvacrol, *p*-cymene/*β*-Myrcene, *α*-phellandrene	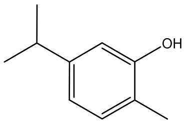	DPPH	[50]
Marigold/ Oregano	*Calendula officinalis/ Origanum compactum*	*α*-Cadinene, δ-cadinene/*β*-myrcene, *α*-phellandrene	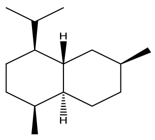	DPPH	[50]

Marigold/ Thyme	*Calendula officinalis*/ *Thymus leptobotrys*	α-Cadinene, δ-cadinene/carvacrol, p-cymene	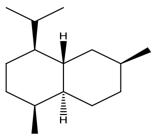	DPPH	[50]
Cinnamon/Clove	*Cinnamomum zeylancium*/*Syzygium aromaticum*	Cinnamaldehyde, linalool/eugenol, eugenol acetate	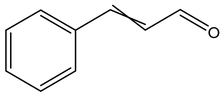	DPPH	[51]
Sweet olive/ Green tea	*Osmanthus fragrans*/ Green tea	Acteoside, salidroside/caffeine, gallic acid	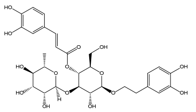	DPPH	[52]
Oregano/ Ajowan	*Oreganum vulgare*/ *Trachyspermum ammi*	Carvacrol, thymol/γ-terpinene, *p*-cymene	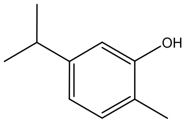	DPPH, BCB	[53]
Ajowan/ Borage	*Trachyspermum ammi*/ *Plectranthus amboinicus*	γ-Terpinene, *p*-cymene/carvacrol, γ-terpinene	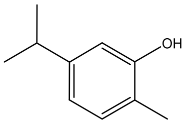	DPPH, BCB	[53]
Borage/ Oregano	*Plectranthus Amboinicus*/ *Oreganum vulgare*	Carvacrol, γ-terpinene/Carvacrol, thymol	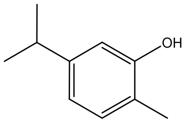	DPPH, BCB	[53]
Oregano/ Ajowan/ Borage	*Oreganum vulgare*/ *Trachyspermum ammi*/ *Plectranthus amboinicus*	Carvacrol, thymol/γ-terpinene, *p*-cymene/carvacrol, γ-terpinene	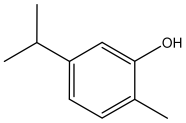	DPPH, BCB	[53]
Lemon myrtle/Kakadu plum/Finger lime/Australian sprouts	*Backhousia citriodora*/ *Terminalia ferdinandiana*/*Citrus australasica*/ *Lophopyrum ponticum*	Geranial, neral/linoleic acid, oleic acid/bicyclogermacrene, α-pinene	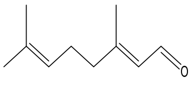	DPPH	[54]

Table S4. Combination of different essential oils with major constituents and their antibacterial interactions against various microorganisms.

Essential Oils	Plant Source	Major Constituents	Molecular Structure	Microbial Strains	Ref.

Lavender/ Marjoram	*Lavandula dentata/ Origanum majorana*	Terpinene-4-ol, trans-4-thujanol/*β*-pinene, 1,8-cineole	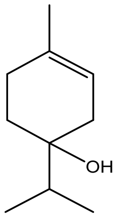	*S. aureus, E. coli*	[55]
Thyme/ Marjoram	*Thymus serpyllum/ Origanum majorana*	*p*-Cymene and *γ*-terpinene/*β*-pinene and 1,8-cineole	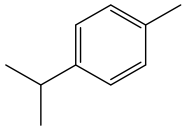	*S. aureus, E. coli*	[55]
Fingerroot/ Basil	*Boesenbergia pandurate /Ocimum sanctum*	Camphor, 1,8-cineole/ eugenol, methyl-eugenol	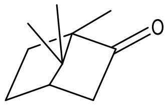	*L. monocytogenes, S. aureus, E. coli, S. enterica*	[56]
Basil/ Lemongrass	*Ocimum sanctum/Cymbopogon citratus*	Eugenol, methyl-eugenol/citral	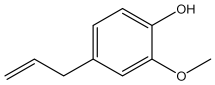	*E. coli, S. enterica*	[56]
Fingerroot/ Lemongrass	*Boesenbergia pandurate /Cymbopogon citratus*	Camphor, 1,8-cineole/ citral	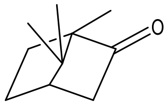	*S. enterica*	[56]
Thyme/ Parsley	*Thymus capitatus/ Petroselimum sativum*	*p*-Cymene/myristicin	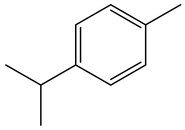	*L. monocytogenes*	[57]
Thyme/ Cumin	*Thymus capitatus/ Cuminun cyminum*	*p*-Cymene/cuminaldehyde, *p*-cymene	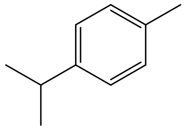	*S. aureus*	[57]
Thyme/Garlic	*Thymus capitatus/ Allium sativum*	*p*-cymene/diallyl disulfide	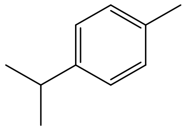	*S. aureus*	[57]
Lavender/ Camphor	*Lavandula latifolia/ Cinnamonum camphora*	Linalool, 1,8-cineole/camphor, linalool	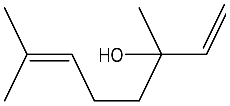	*L. monocytogenes, S. aureus*	[58]
Coriander/ Cumin	*Coriandrum sativum/Cuminum cyminum*	Linalool, *p*-coumaric acid/cuminaldehyde, *p*-cymene	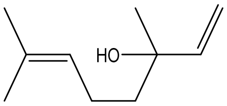	*S. typhimurium/M. Luteus*	[59]

Black pepper/ Cumin/Cinnamon/Turmeric	*Piper nigrum/Cuminum cyminum/Cinnamomum verum/Curcuma longa*	Limonene/cuminaldehyde/eugenol/β-Turmerone	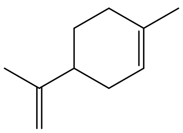	*S. epidermidis*	[60]
Black pepper/Cinnamon	*Piger nigerium/Cinnamomum zeylancium*	α-Pinene, β-pinene/cinnamaldehyde, linalool	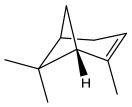	*L. monocytogenes, S. aureus, S. typhimurium*	[61]
Black pepper/Clove	*Piper nigrum/Syzygium aromaticum*	α-Pinene, β-pinene/eugenol, β-caryophyllene	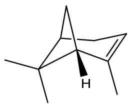	*L. monocytogenes, S. aureus, S. typhimurium*	[61]
Cinnamon/Clove	*Cinnamomum zeylancium/Syzygium aromaticum*	Cinnamaldehyde, linalool/eugenol, eugenol acetate	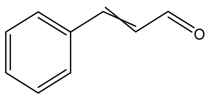	*L. monocytogenes, S. aureus, S. typhimurium*	[61]
Cinnamon/ Oregano	*Cinnamomum zeylanicum/Origanum vulgare*	Cinnamaldehyde, linalool/carvacrol, *p*-cymene	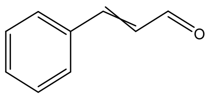	*S. aureus*	[62]
Oregano/ Rosemary	*Origanum vulgare/ Rosmarinus officinalis*	Carvacrol, *p*-cymene/ 1,8-cineole, camphor	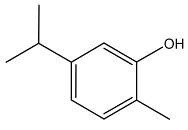	*L. monocytogenes, Y. enterocolitica, A. hydrophilla.*	[63]
Lippia/ Peppermint	*Lippia multiflora/ Mentha piperita*	*p*-Cymene, β-caryophyllene/menthol, menthone	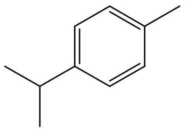	*E. coli, E. aerogenes*	[64]
Clove/ Rosemary	*Syzygium aromaticum/ Rosmarinus officinalis*	Eugenol, β-caryophyllene/1,8-cineole, α-pinene	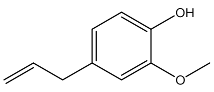	*C. albicans*	[65]

References

1. Kumar, V.; Mathela, C.S.; Kumar, M.; Tewari, G. Antioxidant potential of essential oils from some Himalayan Asteraceae and Lamiaceae species. *Med. Drug Discov.* **2019**, *1*, 100004, doi:10.1016/j.medidd.2019.100004.
2. Olmedo, R.; Ribotta, P.; Grosso, N.R. Antioxidant Activity of Essential Oils Extracted from Aloysia triphylla and Minthostachys mollis that Improve the Oxidative Stability of Sunflower Oil under Accelerated Storage Conditions . *Eur. J. Lipid Sci. Technol.* **2018**, *120*, 1700374, doi:10.1002/ejlt.201700374.
3. Olivas, N.A.; Bejarano, C.V.; Soto, G.A.; Ortega, M.Z.; Salas, F.S.; Chávez, E.S.; Ochoa, L.H. Bioactive compounds and antioxidant activity of essential oils of Origanum dictamnus from Mexico. *AIMS Agric. Food* **2020**, *5*, 387–394, doi:10.3934/AGRFOOD.2020.3.387.
4. Benyoucef, F.; El Amine Dib, M.; Arrar, Z.; Costa, J.; Muselli, A. Synergistic antioxidant activity and chemical composition of essential oils from thymus fontanesii, artemisia herba-alba and rosmarinus officinalis. *J. Appl. Biotechnol. Reports* **2018**, *5*, 151–156, doi:10.29252/JABR.05.04.03.
5. Purkait, S.; Bhattacharya, A.; Bag, A.; Chattopadhyay, R.R. Antibacterial and antioxidant potential of Essential Oils of five spices. *J. Food Qual. Hazards Control* **2018**, *5*, 61–71, doi:10.29252/jfqhc.5.2.6.

6. Ibáñez, M.D.; López-Gresa, M.P.; Lisón, P.; Rodrigo, I.; Bellés, J.M.; González-Mas, M.C.; Blázquez, M.A. Essential Oils as Natural Antimicrobial and Antioxidant Products in the Agrifood Indus. *Nereis. Interdiscip. Ibero-American J. Methods, Model. Simulation.* **2020**, 55–69, doi:10.46583/nereis_2020.12.585.
7. Ben Hsouna, A.; Ben Halima, N.; Smaoui, S.; Hamdi, N. Citrus lemon essential oil: Chemical composition, antioxidant and antimicrobial activities with its preservative effect against Listeria monocytogenes inoculated in minced beef meat. *Lipids Health Dis.* **2017**, *16*, 1–11, doi:10.1186/s12944-017-0487-5.
8. He, T.; Li, X.; Wang, X.; Xu, X.; Yan, X.; Li, X.; Sun, S.; Dong, Y.; Ren, X.; Liu, X.; et al. Chemical composition and anti-oxidant potential on essential oils of Thymus quinquecostatus Celak. from Loess Plateau in China, regulating Nrf2/Keap1 signaling pathway in zebrafish. *Sci. Rep.* **2020**, *10*, 1–18, doi:10.1038/s41598-020-68188-8.
9. Patra, J.K.; Das, G.; Baek, K.H. Chemical composition and antioxidant and antibacterial activities of an essential oil extracted from an edible seaweed, Laminaria japonica L. *Molecules* **2015**, *20*, 12093–12113, doi:10.3390/molecules200712093.
10. Bouyahya, A.; Lagrouh, F.; El Omari, N.; Bourais, I.; El Jemli, M.; Marmouzi, I.; Salhi, N.; Faouzi, M.E.A.; Belmehdi, O.; Dakka, N.; et al. Essential oils of Mentha viridis rich phenolic compounds show important antioxidant, antidiabetic, dermatoprotective, antidermatophyte and antibacterial properties. *Biocatal. Agric. Biotechnol.* **2020**, *23*, 101471, doi:10.1016/j.bcab.2019.101471.
11. Ahmed, A.F.; Attia, F.A.K.; Liu, Z.; Li, C.; Wei, J.; Kang, W. Antioxidant activity and total phenolic content of essential oils and extracts of sweet basil (Ocimum basilicum L.) plants. *Food Sci. Hum. Wellness* **2019**, *8*, 299–305, doi:10.1016/j.fshw.2019.07.004.
12. Zhang, X.; Guo, Y.; Guo, L.; Jiang, H.; Ji, Q. In vitro evaluation of antioxidant and antimicrobial activities of melaleuca alternifolia essential oil. *Biomed Res. Int.* **2018**, *2018*, doi:10.1155/2018/2396109.
13. Guo, J. jing; Gao, Z. peng; Xia, J. lan; Ritenour, M.A.; Li, G. yang; Shan, Y. Comparative analysis of chemical composition, antimicrobial and antioxidant activity of citrus essential oils from the main cultivated varieties in China. *Lwt* **2018**, *97*, 825–839, doi:10.1016/j.lwt.2018.07.060.
14. Avanço, G.B.; Ferreira, F.D.; Bomfim, N.S.; Peralta, R.M.; Brugnari, T.; Mallmann, C.A.; de Abreu Filho, B.A.; Mikcha, J.M.G.; Machinski Jr, M. Curcuma longa L. essential oil composition, antioxidant effect, and effect on Fusarium verticillioides and fumonisin production. *Food Control* **2017**, *73*, 806–813.
15. Bączek, K.B.; Kosakowska, O.; Przybył, J.L.; Pióro-Jabrucka, E.; Costa, R.; Mondello, L.; Gniewosz, M.; Synowiec, A.; Węglarz, Z. Antibacterial and antioxidant activity of essential oils and extracts from costmary (Tanacetum balsamita L.) and tansy (Tanacetum vulgare L.). *Ind. Crops Prod.* **2017**, *102*, 154–163, doi:10.1016/j.indcrop.2017.03.009.
16. Golkar, P.; Mosavat, N.; Jalali, S.A.H. Essential oils, chemical constituents, antioxidant, antibacterial and in vitro cytotoxic activity of different Thymus species and Zataria multiflora collected from Iran. *South African J. Bot.* **2020**, *130*, 250–258, doi:10.1016/j.sajb.2019.12.005.
17. Snuossi, M.; Trabelsi, N.; Taleb, S. Ben; Dehmeni, A.; Flamini, G.; De Feo, V. Laurus nobilis, Zingiber officinale and Anethum graveolens essential oils: Composition, antioxidant and antibacterial activities against bacteria isolated from fish and shellfish. *Molecules* **2016**, *21*, doi:10.3390/molecules21101414.
18. Stanojevic, L.P.; Stanojevic, J.S.; Savic, V.L.; Cvetkovic, D.J.; Kolarevic, A.; Marjanovic-Balaban, Z.; Nikolic, L.B. Peppermint and Basil Essential Oils: Chemical Composition, in vitro Antioxidant Activity and in vivo Estimation of Skin Irritation. *J. Essent. Oil-Bearing Plants* **2019**, *22*, 979–993, doi:10.1080/0972060X.2019.1661793.
19. Hashemi, M.; Ehsani, A.; Hassani, A.; Afshari, A.; Aminzare, M.; Sahranavard, T.; Azimzadeh, Z. Phytochemical, Antibacterial, Antifungal and Antioxidant Properties of Agastache foeniculum Essential Oil. *J. Chem. Heal. Risks* **2017**, *7*, 95–104.
20. Behbahani, B.A.; Shahidi, F.; Yazdi, F.T.; Mortazavi, S.A.; Mohebbi, M. Antioxidant activity and antimicrobial effect of tarragon (Artemisia dracunculus) extract and chemical composition of its essential oil. *J. Food Meas. Charact.* **2017**, *11*, 847–863, doi:10.1007/s11694-016-9456-3.
21. Hailu, Y.M.; Atlabachew, M.; Chandravanshi, B.S.; Redi-Abshiro, M. Composition of essential oil and antioxidant activity of Khat (Catha edulis Forsk), Ethiopia. *Chem. Int* **2017**, *3*, 25.
22. Ouedrhiri, W.; Balouiri, M.; Bouhdid, S.; Harki, E.H.; Moja, S.; Greche, H. Antioxidant and antibacterial activities of Pelargonium asperum and Ormenis mixta essential oils and their synergistic antibacterial effect. *Environ. Sci. Pollut. Res.* **2018**, *25*, 29860–29867, doi:10.1007/s11356-017-9739-1.

23. Ksouda, G.; Sellimi, S.; Merlier, F.; Falcimaigne-cordin, A.; Thomasset, B.; Nasri, M.; Hajji, M. Composition, antibacterial and antioxidant activities of Pimpinella saxifraga essential oil and application to cheese preservation as coating additive. *Food Chem.* **2019**, *288*, 47–56, doi:10.1016/j.foodchem.2019.02.103.
24. Smeriglio, A.; Denaro, M.; Barreca, D.; Calderaro, A.; Bisignano, C.; Ginestra, G.; Bellocco, E.; Trombetta, D. In vitro evaluation of the antioxidant, cytoprotective, and antimicrobial properties of essential oil from Pistacia vera L. Variety Bronte Hull. *Int. J. Mol. Sci.* **2017**, *18*, doi:10.3390/ijms18061212.
25. Bahadori, M.B.; Zengin, G.; Bahadori, S.; Maggi, F.; Dinparast, L. Chemical composition of essential oil, antioxidant, antidiabetic, anti-obesity, and neuroprotective Properties of Prangos gaubae. *Nat. Prod. Commun.* **2017**, *12*, 1945–1948, doi:10.1177/1934578x1701201233.
26. Scur, M.C.; Pinto, F.G.S.; Pandini, J.A.; Costa, W.F.; Leite, C.W.; Temponi, L.G. Antimicrobial and antioxidant activity of essential oil and different plant extracts of Psidium cattleianum Sabine. *Brazilian J. Biol.* **2016**, *76*, 101–108, doi:10.1590/1519-6984.13714.
27. Luís, Â.; Duarte, A.; Gominho, J.; Domingues, F.; Duarte, A.P. Chemical composition, antioxidant, antibacterial and anti-quorum sensing activities of Eucalyptus globulus and Eucalyptus radiata essential oils. *Ind. Crops Prod.* **2016**, *79*, 274–282, doi:10.1016/j.indcrop.2015.10.055.
28. Man, A.; Santacroce, L.; Jacob, R.; Mare, A.; Man, L. Antimicrobial activity of six essential oils against a group of human pathogens: A comparative study. *Pathogens* **2019**, *8*, 1–11, doi:10.3390/pathogens8010015.
29. Tardugno, R.; Serio, A.; Pellati, F.; D'Amato, S.; Chaves López, C.; Bellardi, M.G.; Di Vito, M.; Savini, V.; Paparella, A.; Benvenuti, S. Lavandula x intermedia and Lavandula angustifolia essential oils: phytochemical composition and antimicrobial activity against foodborne pathogens. *Nat. Prod. Res.* **2019**, *33*, 3330–3335, doi:10.1080/14786419.2018.1475377.
30. Kapp, K.; Püssa, T.; Orav, A.; Roasto, M.; Raal, A.; Vuorela, P.; Vuorela, H.; Tammela, P. Chemical Composition and Antibacterial Effect of Mentha spp. Grown in Estonia. *Nat. Prod. Commun.* **2020**, *15*, 1–14, doi:10.1177/1934578X20977615.
31. Condò, C.; Anacarso, I.; Sabia, C.; Iseppi, R.; Anfelli, I.; Forti, L.; de Niederhäusern, S.; Bondi, M.; Messi, P. Antimicrobial activity of spices essential oils and its effectiveness on mature biofilms of human pathogens. *Nat. Prod. Res.* **2020**, *34*, 567–574, doi:10.1080/14786419.2018.1490904.
32. Mekonnen, A.; Yitayew, B.; Tesema, A.; Taddese, S. In Vitro Antimicrobial Activity of Essential Oil of Thymus schimperi, Matricaria chamomilla, Eucalyptus globulus, and Rosmarinus officinalis. *Int. J. Microbiol.* **2016**, *2016*, doi:10.1155/2016/9545693.
33. Wei, Z.F.; Zhao, R.N.; Dong, L.J.; Zhao, X.Y.; Su, J.X.; Zhao, M.; Li, L.; Bian, Y.J.; Zhang, L.J. Dual-cooled solvent-free microwave extraction of Salvia officinalis L. essential oil and evaluation of its antimicrobial activity. *Ind. Crops Prod.* **2018**, *120*, 71–76, doi:10.1016/j.indcrop.2018.04.058.
34. Kabir Mumu, S.; Mahboob Hossain, M. Antimicrobial Activity of Tea Tree oil against Pathogenic Bacteria and Comparison of Its Effectiveness with Eucalyptus Oil, Lemongrass Oil and Conventional Antibiotics. *Am. J. Microbiol. Res.* **2018**, *6*, 73–78, doi:10.12691/ajmr-6-3-2.
35. Khalil, N.; Ashour, M.; Fikry, S.; Naser, A.; Salama, O. Future Journal of Pharmaceutical Sciences Chemical composition and antimicrobial activity of the essential oils of selected Apiaceous fruits. *Futur. J. Pharm. Sci.* **2018**, *4*, 88–92, doi:10.1016/j.fjps.2017.10.004.
36. Cazella, L.N.; Glamoclija, J.; Soković, M.; Gonçalves, J.E.; Linde, G.A.; Colauto, N.B.; Gazim, Z.C. Antimicrobial activity of essential oil of Baccharis dracunculifolia DC (Asteraceae) aerial parts at flowering period. *Front. Plant Sci.* **2019**, *10*, 27.
37. Helal, I.M.; El-bessoumy, A.; Al-bataineh, E.; Joseph, M.R.P.; Rajagopalan, P.; Chandramoorthy, H.C.; Ben, S.; Ahmed, H. Antimicrobial Efficiency of Essential Oils from Traditional Medicinal Plants of Asir Region , Saudi Arabia , over Drug Resistant Isolates. **2019**, *2019*, doi:10.1155/2019/8928306.
38. Moussii, I.M.; Nayme, K.; Timinouni, M.; Jamaleddine, J.; Filali, H.; Hakkou, F. Synergistic antibacterial effects of Moroccan Artemisia herba alba, Lavandula angustifolia and Rosmarinus officinalis essential oils. *Synergy* **2020**, *10*, 100057.
39. Mutlu-Ingok, A.; Tasir, S.; Seven, A.; Akgun, N.; Karbancioglu-Guler, F. Evaluation of the single and combined antibacterial efficiency of essential oils for controlling Campylobacter coli, Campylobacter jejuni, Escherichia coli, Staphylococcus aureus, and mixed cultures. *Flavour Fragr. J.* **2019**, *34*, 280–287.
40. Tu, X.-F.; Hu, F.; Thakur, K.; Li, X.-L.; Zhang, Y.-S.; Wei, Z.-J. Comparison of antibacterial effects and fumigant toxicity of essential oils extracted from different plants. *Ind. Crops Prod.* **2018**, *124*, 192–200.

41. Gishen, N.Z.; Taddese, S.; Zenebe, T.; Dires, K.; Tedla, A.; Mengiste, B.; Shenkute, D.; Tesema, A.; Shiferaw, Y.; Lulekal, E. In vitro antimicrobial activity of six Ethiopian medicinal plants against Staphylococcus aureus, Escherichia coli and Candida albicans. *Eur. J. Integr. Med.* **2020**, *36*, 101121.
42. Bouyahya, A.; Et-Touys, A.; Bakri, Y.; Talbaui, A.; Fellah, H.; Abrini, J.; Dakka, N. Chemical composition of Mentha pulegium and Rosmarinus officinalis essential oils and their antileishmanial, antibacterial and antioxidant activities. *Microb. Pathog.* **2017**, *111*, 41–49.
43. Abou Baker, D.H.; Al-Moghazy, M.; ElSayed, A.A.A. The in vitro cytotoxicity, antioxidant and antibacterial potential of Satureja hortensis L. essential oil cultivated in Egypt. *Bioorg. Chem.* **2020**, *95*, 103559.
44. Imane, N.I.; Fouzia, H.; Azzahra, L.F.; Ahmed, E.; Ismail, G.; Idrissa, D.; Mohamed, K.-H.; Sirine, F.; L'Houcine, O.; Noureddine, B. Chemical composition, antibacterial and antioxidant activities of some essential oils against multidrug resistant bacteria. *Eur. J. Integr. Med.* **2020**, *35*, 101074.
45. Aumeeruddy-Elalfi, Z.; Gurib-Fakim, A.; Mahomoodally, F. Antimicrobial, antibiotic potentiating activity and phytochemical profile of essential oils from exotic and endemic medicinal plants of Mauritius. *Ind. Crops Prod.* **2015**, *71*, 197–204.
46. Radaelli, M.; Silva, B.P. da; Weidlich, L.; Hoehne, L.; Flach, A.; Costa, L.A.M.A. da; Ethur, E.M. Antimicrobial activities of six essential oils commonly used as condiments in Brazil against Clostridium perfringens. *brazilian J. Microbiol.* **2016**, *47*, 424–430.
47. Hyldgaard, M.; Mygind, T.; Meyer, R.L. Essential oils in food preservation: mode of action, synergies, and interactions with food matrix components. *Front. Microbiol.* **2012**, *3*, 12.
48. Benyoucef, F.; Dib, M.E.A.; Arrar, Z.; Costa, J.; Muselli, A. Synergistic antioxidant activity and chemical composition of essential oils from Thymus fontanesii, Artemisia herba-alba and Rosmarinus officinalis. *J. Appl. Biotechnol. Reports* **2018**, *5*, 151–156.
49. Crespo, Y.A.; Sánchez, L.R.B.; Quintana, Y.G.; Cabrera, A.S.T.; Del Sol, A.B.; Mayancha, D.M.G. Evaluation of the synergistic effects of antioxidant activity on mixtures of the essential oil from Apium graveolens L., Thymus vulgaris L. and Coriandrum sativum L. using simplex-lattice design. *Heliyon* **2019**, *5*, e01942.
50. Bounimi, S.; Chebli, B. Synergistic antioxidant activity of three essential oils of Lamiacea family from Morocco. *Appl. J. Environ. Eng. Sci.* **2017**, *3*, 195–200.
51. Purkait, S.; Bhattacharya, A.; Bag, A.; Chattopadhyay, R.R. Synergistic antibacterial, antifungal and antioxidant efficacy of cinnamon and clove essential oils in combination. *Arch. Microbiol.* **2020**, 1–10.
52. Mao, S.; Wang, K.; Lei, Y.; Yao, S.; Lu, B.; Huang, W. Antioxidant synergistic effects of Osmanthus fragrans flowers with green tea and their major contributed antioxidant compounds. *Sci. Rep.* **2017**, *7*, 1–10.
53. Hafeeza, K.; Kulathooran, R.; Pullabhatla, S.; Babasaheb Bhaskarrao, B. Synergistic antioxidant action of oregano, ajowan and borage extracts. *Food Nutr. Sci.* **2011**, *2011*.
54. Shami, A.-M.M.; Philip, K.; Muniandy, S. Synergy of antibacterial and antioxidant activities from crude extracts and peptides of selected plant mixture. *BMC Complement. Altern. Med.* **2013**, *13*, 1–11.
55. Ouedrhiri, W.; Mounyr, B.; Harki, E.H.; Moja, S.; Greche, H. Synergistic antimicrobial activity of two binary combinations of marjoram, lavender, and wild thyme essential oils. *Int. J. food Prop.* **2017**, *20*, 3149–3158.
56. Tantala, J.; Rachtanapun, P.; Rachtanapun, C. Synergistic Antimicrobial Activities of Thai Household Essential Oils in Chitosan Film. *Polymers (Basel).* **2021**, *13*, 1519.
57. García-Díez, J.; Alheiro, J.; Pinto, A.L.; Falco, V.; Fraqueza, M.J.; Patarata, L. Synergistic activity of essential oils from herbs and spices used on meat products against food borne pathogens. *Nat. Prod. Commun.* **2017**, *12*, 1934578X1701200236.
58. Karaca, N.; Şener, G.; Demirci, B.; Demirci, F. Synergistic antibacterial combination of Lavandula latifolia Medik. essential oil with camphor. *Zeitschrift für Naturforsch. C* **2020**, *1*.
59. Bag, A.; Chattopadhyay, R.R. Evaluation of synergistic antibacterial and antioxidant efficacy of essential oils of spices and herbs in combination. *PLoS One* **2015**, *10*, e0131321.
60. Jardak, M.; Mnif, S.; Ayed, R. Ben; Rezgui, F.; Aifa, S. Chemical composition, antibiofilm activities of Tunisian spices essential oils and combinatorial effect against Staphylococcus epidermidis biofilm. *LWT* **2021**, *140*, 110691.
61. Purkait, S.; Bhattacharya, A.; Bag, A.; Chattopadhyay, R.R. Synergistic antibacterial, antifungal and antioxidant efficacy of cinnamon and clove essential oils in combination. *Arch. Microbiol.* **2020**, *202*, 1439–1448, doi:10.1007/s00203-020-01858-3.

62. Doi, N.M.; Sae-Eaw, A.; Suppakul, P.; Chompreeda, P. Assessment of synergistic effects on antimicrobial activity in vapour-and liquid-phase of cinnamon and oregano essential oils against Staphylococcus aureus. *Int. Food Res. J.* **2019**, *26*, 459–467.
63. De Azeredo, G.A.; Stamford, T.L.M.; Nunes, P.C.; Neto, N.J.G.; De Oliveira, M.E.G.; De Souza, E.L. Combined application of essential oils from Origanum vulgare L. and Rosmarinus officinalis L. to inhibit bacteria and autochthonous microflora associated with minimally processed vegetables. *Food Res. Int.* **2011**, *44*, 1541–1548.
64. Bassolé, I.H.N.; Lamien-Meda, A.; Bayala, B.; Tirogo, S.; Franz, C.; Novak, J.; Nebié, R.C.; Dicko, M.H. Composition and antimicrobial activities of Lippia multiflora Moldenke, Mentha x piperita L. and Ocimum basilicum L. essential oils and their major monoterpene alcohols alone and in combination. *Molecules* **2010**, *15*, 7825–7839.
65. Fu, Y.; Zu, Y.; Chen, L.; Shi, X.; Wang, Z.; Sun, S.; Efferth, T. Antimicrobial activity of clove and rosemary essential oils alone and in combination. *Phyther. Res.* **2007**, *21*, 989–994.
